# Photocatalytic Transformations of 1H-Benzotriazole and Benzotriazole Derivates

**DOI:** 10.3390/nano10091835

**Published:** 2020-09-14

**Authors:** Marco Minella, Elisa De Laurentiis, Francesco Pellegrino, Marco Prozzi, Federica Dal Bello, Valter Maurino, Claudio Minero

**Affiliations:** 1 Chemistry Department and NIS Interdepartmental Centre, University of Torino, Via P. Giuria 5, 10125 Turin, Italy; marco.minella@unito.it (M.M.); elisa.delaurentiis@tiscali.it (E.D.L.); francesco.pellegrino@unito.it (F.P.); marco.prozzi@unito.it (M.P.); claudio.minero@unito.it (C.M.); 2 JointLAB UniTo-ITT Automotive, Via Quarello 15/A, 10135 Torino, Italy; 3 Department of Molecular Biotechnology and Health Sciences, University of Torino, Via P. Giuria 5, 10125 Turin, Italy; federica.dalbello@unito.it

**Keywords:** photocatalysis, 1H-benzotriazole, tolyltriazole, Tinuvin P, emerging pollutants, titanium dioxide

## Abstract

Benzotriazoles are a new class of organic emerging pollutants ubiquitously found in the environment. The increase of their concentration to detectable values is the consequence of the inability of the Conventional Waste Water Plants (CWWPs) to abate these products. We subjected 1H-benzotriazole (BTz), tolyltriazole (TTz), and Tinuvin P (TP, a common UV plastic stabilizer) to photocatalytic degradation under UV-irradiated TiO_2_ in different conditions. The principal photoformed intermediates, the relationship between the degradation rate and the pH, the degree of mineralization, and the fate of the organic nitrogen were investigated. Under the adopted experimental conditions, all the studied substrates were rapidly photocatalytically transformed (the maximum degradation rates for BTz and TTz were (3.88 ± 0.05) × 10^−2^ and (2.11 ± 0.09) × 10^−2^ mM min^−1^, respectively) and mineralized (the mineralization rate for BTz and TTz was 4.0 × 10^−3^ mM C min^−1^ for both substrates). Different from the 1,2,4-triazole rings that are not completely mineralized under photocatalytic conditions, 1H-benzotriazole and tolyltriazole were completely mineralized with a mechanism that involved a partial conversion of organic nitrogen to N_2_. The photocatalytic process activated by UV-irradiated TiO_2_ is an efficient tool to abate 1H-benzotriazole and its derivatives, avoiding their release in the environment.

## 1. Introduction

Human activities (e.g., industrial processes and agricultural productions) release in the environment xenobiotic compounds that are often not biodegradable and not removed by the Conventional Waste Water Plants (CWWPs). The long environmental residence time of these molecules causes their accumulation until detectable levels in the environment and often their biomagnification in the food chain. CECs (Contaminants of Emerging Concern), POPs (Persistent Organic Pollutants), GOCs (Global Organic Contaminants), and EDCs (Endocrine Disrupting Chemicals) are only some of the acronyms used to identify new classes of emerging pollutants [1].

Benzotriazoles find broad application in industrial processes and in everyday uses. The most used compounds of this class are 1H-benzotriazole (BTz) and tolyltriazole (a mixture of 4-methyl- and 5-methyl-1H-benzotriazole, TTz). These chemicals show strong complexing properties and are used as anticorrosive agents in cooling, hydraulic, and antifreezing fluids; moreover, they are additives in washing powders containing detergents or bleaching agents. Moreover, BTz and TTz are chemical intermediates in the synthesis of dyes, drugs, and fungicides. The derivates of BTz with a phenolic group in position 2, commercialized with the name Tinuvin, are used as UV stabilizers in plastic materials [2]. BTz and TTz are also constituents of Aircraft Deicer and Anti-Icer fluids (ADAFs) widely used for aircrafts and airport runways. Airports are hot spots for the release of these compounds into the environment. The total production of benzotriazoles can be estimated from 9000 tons/year worldwide to 9000 tons/year for the United States of America only [3].

BTz and TTz are weak organic acids with pK_a_ equal to 8.6 and 8.8, respectively, with high water solubility (28 and 7 g dm^−3^, respectively), low vapor pressure, and low octanol water distribution coefficients (logK_ow_: 1.23 and 1.89, respectively) [4,5].

Cancilla et al. [6] demonstrated that the mixture of BTz and TTz is the primary cause of toxicity in ADAFs. Some reports focused on the toxicity of BTz and TTz classified these compounds as “toxic to aquatic organisms and a cause of long-term effects in the aquatic environment” [7,8]. Harris et al. [9] demonstrated that BTz has clear antiestrogenic effect properties in vitro, but the subsequent in vivo studies showed no evidence of antiestrogenic activity. Harris’ conclusion is that “a rigorous investigation of the chronic toxicity of BTz is imperative” [9]. An overall evaluation of the occurrence, toxicity, and transformation of the six benzotriazoles most detected in the environment has been recently reported by Shi et al. [10].

Benzotriazoles are assumed as not biodegradable compounds. To degrade these molecules, the microorganisms have to cleave the aromatic ring, but the enzymes that are able to hydroxylate the aromatic ring, promoting their cleavage (e.g., cytocrome P450), are deactivated by benzotriazoles [11]. Consequently, the CWWPs have low ability to remove benzotriazoles [12,13,14,15]. Giger and co-workers demonstrated that BTz and TTz are ubiquitous contaminants in the aquatic environment. They found detectable concentrations of BTz and TTz in some Swiss streams and lakes (with a maximum BTz concentration of 6.3 µg dm^−3^) [16]. The presence of BTz and TTz in the effluents of municipal wastewater plants was observed [12,17]. The highest concentrations of benzotriazoles were detected on water sampled during the winter period because of the large use of benzotriazoles in ADAFs [16,18].

To avoid the environment release of benzotriazoles, Weiss et al. [12] proposed the treatment of municipal wastewater by membrane bioreactors (MBR), which increase the removal ability of the wastewater plants, but could not avoid a relevant release of benzotriazoles in the plant effluents. The complete removal of these compounds was obtained only by ozonation of the water, allowing an almost complete degradation of BTz and TTz [12,19]. The phytotransformation was also proposed for the benzotriazole abatement [20], but this is not a feasible approach for the treatment of high volumes of municipal waste water. The direct photochemical degradation of benzotriazoles was studied [21], and BTz is significantly degraded by UV radiation, but not mineralized: aniline and phenazine were observed as principal and more toxic intermediates [5]. Studies regarding the photocatalytic degradation of BTz and TTz are scarce. Recently, the photocatalytic processes over irradiated semiconductors have been proposed for the abatement of BTz. Chen et al. compared the transformation rate of benzotriazole by UV/H_2_O_2_ and UV/TiO_2_, investigating the kinetics of transformation and the nature of the main by-products, and they observed the decrease of the toxicity with the progressive disappearance of BTz [22]. The photocatalytic degradation of BTz in the presence of peroxymonosulfate (PMS) and persulfate (PS) as alternative oxidants (conduction band electron scavengers) to oxygen was investigated by Ahmadi et al. With PM and PMS, the rate of BTz transformation increased, and the main oxidant species was the sulfate radical with both oxidants [23]. The use of pristine TiO_2_ film deposited on glassy carbon electrodes for the photoelectrocatalytic oxidation of BTz was investigated by Ding and co-workers. The application of a 0.8 V bias promoted an increase of the BTz degradation mainly minimizing the recombination process between the valence band holes (h^+^_vb_) and conduction band electrons (e^−^_cb_) [24]. Moreover, TiO_2_ functionalized with magnetic activated carbon (Fe^II^Fe_2_^III^O_4_@C) [25] and Cu-modified nanoporous TiO_2_ [26] were both proposed for the removal of BTz from aqueous phase under irradiation. Alternatively, semiconductors different from titanium dioxide have been proposed for the same aim (e.g., CuMoO_4_ [27] and BiOI [28] under visible light and BiOBr under simulated solar irradiation [29]).

Moreover, the photocatalytic degradation of 1-H-1,2,4-triazole and of its derivates 3-amino-1-H-1,2,4-triazole (amitrole) and 3,5-diamino-1-H-1,2,4-triazole (recognized as EDAs), compounds similar to BTz and TTz (note the different position of nitrogen atoms in the triazole ring), was carried out by Watanabe and co-workers [30]. The photomineralization of these compounds was low, and the retention of nitrogen in the intermediates was reported as a consequence of the formation of cyanuric acid as a last by-product, which is a well-noted stable molecule in photocatalytic conditions [31,32]. The photocatalytic degradation of cyanuric acid was photocatalytically obtained under surface-platinized titanium dioxide, as reported by Macyk and co-workers [33].

**Figure 1:**
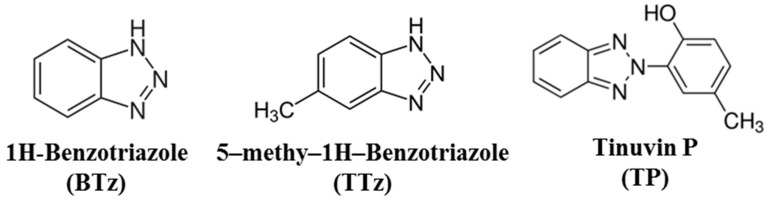
Chemical structures of the investigated substrates.

The aim of this work was to study the photocatalytic degradation under UV-irradiated TiO_2_ of 1H-benzotriazole (BTz), tolyltriazole (TTz, in this work the 5-methyl-1H-benzotriazole isomer) and Tinuvin P (TP, a common UV stabilizer used in plastic materials belonging to the benzotriazole class) (see Table S1 of the Supplementary Material for details). The degradation rate of these molecules (chemical structures reported in Figure 1) and the formation of the principal intermediates were investigated. Attention was devoted to the ability of irradiated TiO_2_ to mineralize BTz and TTz and to the fate of the nitrogen atoms in the triazole ring. The aim of the present work is also to give insights into the mechanism of photocatalytic transformation of this class of compounds. For the first time, the fate of the organic nitrogen in these unusual chemical structures has been investigated.

## 2. Materials and Methods

The photodegradation experiments were carried out by using standard glass cells (4.0 cm diameter and 2.3 cm height, cut-off at 295 nm) on 5 cm^−3^ of aqueous suspension containing the desired amount of the photocatalyst powder (0.5 g dm^−3^) and substrate (BTz, TTz, and Tinuvin P, all purchased from Sigma-Aldrich (St. Louis, MO, USA), in different conditions of pH and substrate concentration. The suspensions containing TiO_2_ were prepared by sonication. The pH of the slurries was adjusted with NaOH 1 M (Sigma-Aldrich, St. Louis, MO, USA) or HClO_4_ 1 M (Sigma-Aldrich, St. Louis, MO, USA), as required.

All reagents used were at least of analytical grade and used as received. Water was purified with a MilliQ plus apparatus (TOC (Total Organic Carbon) = 2 ppb, conductivity 18.2 MΩ cm^−1^).

The nanocrystalline TiO_2_ powder used for the photocatalytic degradation experiments was TiO_2_ Degussa P25 (80% anatase/20% rutile, mean particle size 40–50 nm, BET (Brunauer-Emmett-Teller) surface area 50 m^2^ g^−1^ [34,35], plate-like particles with the relevant amount of surface steps, corners, and borders). The photocatalyst were purified from organic and ionic impurities, which can affect the photocatalyst activity [36] by UV irradiation of a water suspension (48 h), followed by several washings until no inorganic ions were detected by ion chromatography; then, the powder was dried at 80 °C for two hours.

A single tolyltriazole photodegradation experiment was carried out in the presence of TiO_2_ Merck (100% anatase, mean particle size 40–300 nm, BET surface area 10 m^2^ g^−1^, mainly roundish and smooth surfaces) in spite of TiO_2_ P25. A detailed high resolution TEM (Transmission Electron Microscopy) and FTIR (Fourier Transform Infrared) characterization of both catalysts was reported elsewhere [35,37], and reports focused on the different reactivity of these two catalysts have been already published [38,39,40]. Similar transformation rates under irradiated TiO_2_ P25 and Merck were observed in the presence of substrates that react mainly through HO^•^-like mediated oxidation by photoformed holes stabilized at the surface (i.e., adsorbed HO^•^), while a strong selectivity of the P25 TiO_2_ was observed toward substrates that were mainly photocatalytically transformed by a direct charge transfer mechanism [40,41,42].

The irradiation was made by a set of three TLD 18W/08 fluorescence lamps (Philips, Eindhoven, Nederland) in standard conditions (30.5 W m^−2^). The lamp spectrum is characterized by an emission band centred at 368 nm with a width at half maximum of 20 nm. The entire apparatus is described elsewhere [31]. During irradiation, the suspensions were magnetically stirred, and the cell temperature was maintained at 30 ± 3 °C. After irradiation, the suspensions were filtered through 0.45 μm cellulose acetate membranes (Millipore HA) and immediately analyzed. The irradiation experiments were replicated at least three times, and a maximum relative standard deviation for the degradation kinetic constant equal to 5% was observed.

The kinetics of 1H-benzotriazole, tolyltriazole, and Tinuvin P degradation was monitored by HPLC-DAD (Hitachi, Tokyo, Japan) L2200, Elite Lachrom, column Lichrospher R100-CH 18/2 (125 mm, 10 mm diameter)). The elution was carried out in isocratic mode with CH_3_CN:H_2_O = 15:85 and CH_3_CN:H_2_O = 70:30 for BTz and Tinuvin P, respectively, while it was carried out in gradient mode for TTz (CH_3_CN:H_2_O = 15:85 for 5 min, gradient until CH_3_CN:H_2_O = 50:50 for 5 min). The retention time of 1H-benzotriazole, tolyltriazole, and Tinuvin P in the adopted chromatographic conditions were 5.0, 8.0, and 7.1 min, respectively. The injection volume was 60 µL.

The evolution of the nitrate and ammonium concentration during the photocatalytic degradation of BTz was monitored by ion chromatography (Dionex, CA, USA) DX 500 equipped with a GP40 gradient pump, ED40 electrochemical detector, Rheodyne 9126 injector with a 50 µL loop, and Dionex Ion Pac AS9-HC column (250 mm, 4 mm diameter) and Dionex ASRS300 4 mm (electrolytically regenerated suppressor) for the nitrate determination and Dionex Ion Pac CS5A column (250 mm, 4 mm diameter) and Dionex CERS 500 4-mm (electrolytically regenerated suppressor) for the ammonium analysis)). The mobile phase was NaHCO_3_ 9 mM and K_2_CO_3_ 4 mM for the anion analysis and methanesulfonic acid 20 mM for cation quantification. The eluent flow was 1 mL min^−1^.

The determination of the Non Purgeable Organic Carbon (NPOC) was carried out with a Skalar FORMACS TC/TN Analyzer.

The accurate molecular weight of the principal intermediates obtained during the photocatalytic degradation of BTz, TTz, and Tinuvin P was determined analyzing the irradiated solution by High performance liquid chromatography—Electrospray tandem mass spectrometry (HPLC-ESI-MS/MS) with a high-resolution MS detector (LTQ-Orbitrap, Thermo Scientific, Waltham, MA, USA). The chromatographic separation was obtained using 0.1% formic acid (purchased from Merck) in water (eluent A) and acetonitrile (eluent B, purchased from Merck, Darmstadt, Germany) in a gradient run from 5% to 100% of eluent B in 18 min. Then, the column went back to the initial condition. The flow rate and injection volume were 200 µL min^−1^ and 20 µL, respectively. The LC column effluent was delivered into the ion source using nitrogen as a sheath and auxiliary gas. The ESI (Electrospray Ionization) positive mode source voltage and the heated capillary were 4.1 kV and 275 °C, respectively. Resolution was set at 30,000 both for full mass and MS^2^ spectra. Full mass spectra were acquired between *m/z* 50 and 500. MS^2^ experiments were obtained in the range between ion trap cut-off and precursor ion *m/z* values. For example, for the ion of 1H-benzotriazole with *m/z* of 120, the range was from *m/z* 50 to 130. The mass accuracy of recorded ions (versus calculated) was ±0.001 u (without internal calibration).

## 3. Results and Discussion

### 3.1. H-Benzotriazole Photodegradation

**Figure 2:**
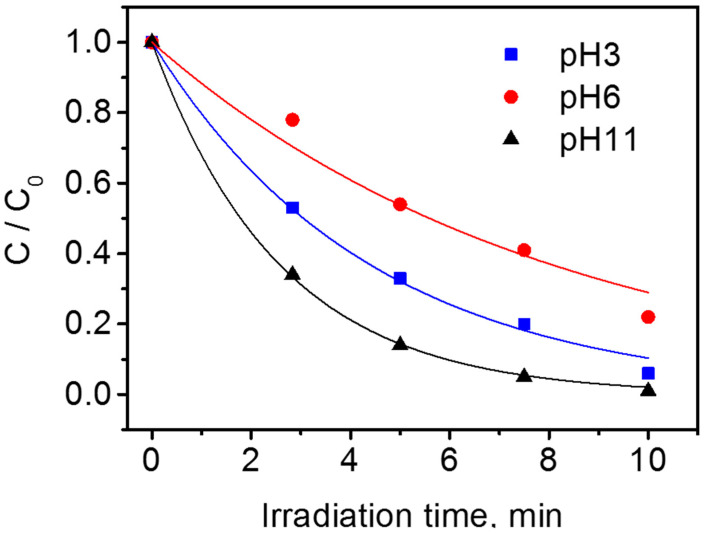
1H-benzotriazole (BTz) photocatalytic degradation as a function of pH (conditions: TiO_2_ P25 0.5 g dm^−3^; BTz initial concentration 1 × 10^−4^ M).

The photodegradation of 1H-benzotriazole was explored at different pHs. Figure 2 shows the profile of BTz degradation at pH 3, 6, and 11.

Due to their extreme stability under UV irradiation, the effect of direct photolysis on the compounds is negligible in the conditions here explored. This peculiar characteristic allows their use as UV absorbers in plastic materials preventing any degradation [43].

The substrate concentration disappearance follows a pseudo-first order kinetic for one to two half-lives. The initial transformation rates are reported in Table S2. An almost complete disappearance of the substrate (initial concentration 1 × 10^−4^ M) was achieved in the first 10–20 min. The rate of a photocatalytic process relies on a complex interplay of physical and chemical parameters. The change of pH can modify drastically the degradation rate due to the change of the position of the semiconductor bands and consequently of the energetics of the photogenerated charges, the hydroxylation degree, the surface charge, the surface adsorption ability, etc.

A non-monotonic trend for the BTz photodegradation rate versus pH was observed. The minimum in the degradation rate was observed at pH 6 (1.2 × 10^−2^ mM min^−1^), while an increase was observed at pH 3 (2.27 × 10^−2^ mM min^−1^) and 11 (3.88 × 10^−2^ mM min^−1^). At the most basic pH, we observed the fastest degradations. It has been reported that the valence band edge potential of TiO_2_ follows Nernstian behavior as a function of pH:

(1) $$E_{\mathrm{VB}}=E_{\mathrm{VB}}\left(\mathrm{pH}=0\right)-\frac{\mathrm{kT}}{\mathrm{elog}\left(10\right)}\mathrm{pH}$$

where E_VB_ (pH = 0) = 3.0 V vs. NHE for TiO_2_ at 298 K [44]. Other authors reported that for surfaces with ionizable groups (clays, inorganic oxides, insoluble salts, latex colloids, etc.), the Nernst equation does not describe accurately the valence band potential as a function of pH, and significant deviations from the theoretical behavior was observed [45]. Moreover, non-Nernstian behaviors have been reported for thin film nanocrystalline TiO_2_ electrodes, especially in the presence of ions strongly adsorbed at the surface [46]. A positive shift of the valence band edge potential, with a consequence increase of the oxidant ability of the photogenerated holes, is undoubtedly a result of the decrease of the solution pH.

The difference in the BTz degradation rate from pH 3 to 6 was not explainable considering the positive shift of the oxidative potential of the photo-formed holes as a consequence of the increase of the H_3_O^+^ activity. The maximum degradation rate was observed at pH 11. This datum was unexplainable, considering the potential of the photo-formed holes. A prominent role of the BTz acid–base equilibrium can be envisaged. At pH 11, the prevalent species for 1H-benzotriazole is the deprotonated form. The experimental evidences show that the deprotonated form of BTz is faster oxidized than the protonated one. At pH 11, a degradation mechanism that involves mainly the oxidation of the triazole ring (the part of the BTz molecule involved in the acid–base equilibrium) as a first step could be hypothesized. Furthermore, at basic pH, the shift at higher energy (lower electrochemical potential) of the conduction band can increase the kinetic of e^−^_cb_ injection toward the dissolved oxygen, promoting an overall increase of the yield of the process as suggested by Cornu et al. to explain the increase of the transformation rate of Methyl Orange at basic pH under UV-irradiated TiO_2_ P25 [47]. Andreozzi and co-workers [21] observed a decrease of the direct photolysis rate increasing the pH as the consequence of the lower molar extinction coefficient of the deprotonated BTz. In the experimental conditions here explored, the role of the direct photolysis is negligible, and the photocatalyzed phenomena prevailed on the direct photolytic ones. The maximum of the photocatalytic degradation rate was observed at basic pH: exactly the opposite of what was observed for the non-catalyzed UV degradation. Note that BTz cannot work as an electron acceptor competing with oxygen for the e^−^_cb_, because the reduction of the benzotriazoles starts at lower potential (roughly −1.3 V vs. NHE [48]) with respect to the potential of the TiO_2_ conduction band (roughly −0.1 V vs. NHE). The lower photocatalytic activity at pH 6 can be also associated to the increased agglomeration that occurs at this pH, which is near the PZC of P25 TiO_2_. The agglomeration of the nanoparticles can reduce the light absorption and the available surface area [49].

**Figure 3:**
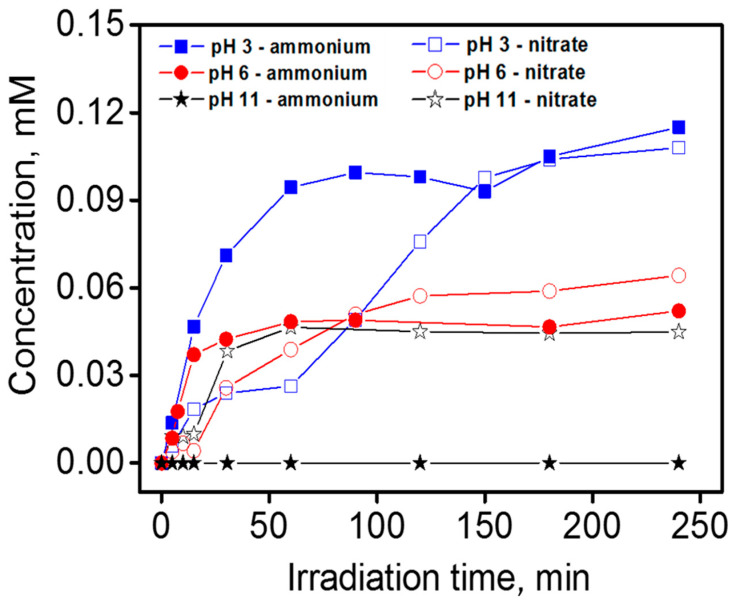
Time profile for nitrate and ammonium during the photocatalytic degradation of 1H-benzotriazole at different pHs (conditions: BTz nominal initial concentration 1 × 10^−4^ M; TiO_2_ P25 0.5 g dm^−3^).

Figure 3 shows the time evolution for the ammonium and nitrate concentration during the photocatalytic degradation of BTz (1 × 10^−4^ M) at pH 3, 6, and 11. The total mineralization of one BTz mole gives 3 moles of inorganic nitrogen. The photomineralization of the substrate in terms of loss of organic nitrogen to NH_4_^+^ and NO_3_^−^ was low. The maximum conversion of the organic nitrogen to ammonium and nitrate was observed at pH 3, where 66% of the initial organic nitrogen was converted to mineral nitrogen. At pH 6, the conversion was lower, and the formation of one nitrate/ammonium ion per two BTz molecules (conversion 33%) was observed. Furthermore, at pH 11, we observed the formation of nitrate, and no ammonium ions were detected (concentration <0.01 mM). At this pH, the lower concentration of ammonium was justified by the loss of ammonium as ammonia for volatilization. This hindered defining an accurate value for the nitrogen conversion at pH 11 (and in general at pH equal to or higher than the pK_b_ of NH_3_).

The defect in the nitrogen balance can be related either to the formation of molecular nitrogen during the degradation of the 1,2,3-triazole ring or to the formation of stable organic compounds contained nitrogen (e.g., cyanuric acid). The latter hypothesis was proposed by Watanabe and co-workers [22] to explain the low nitrogen mineralization observed during the photocatalytic degradation of 1H-1,2,4-triazole and its derivates. Note that urea in photocatalytic conditions forms slowly nitrate and ammonia [50,51].

The organic carbon mineralization of 1H-benzotriazole (1 × 10^−3^ M) was studied observing the evolution of the Non Purgeable Organic Carbon concentration during the photocatalytic degradation at pH 3. The observed trend was reported in Figure S1. A complete mineralization was observed after 240 min of irradiation, and a monotonic decrease of NPOC was observed (mineralization rate = 4.0 × 10^−3^ mM C min^−1^). The absence of intermediate accumulation demonstrates the applicability of the photocatalysis as a possible tool for the complete BTz removal from the aqueous phase. Weiss et al. achieved a high degree of BTz mineralization by the use of Membrane Bioreactor (MB), but with low kinetics, and by the use of a pilot-scale ozonation plant [12]. The complete mineralization of BTz suggested that the defect of nitrogen in the mass balance can be explained with a release of N_2_ during the BTz photocatalytic degradation. The presence in BTz molecules of three nitrogen atoms in contiguous positions (i.e., positions 1, 2, 3) might cause the direct release of N_2_ during the photocatalytic process. During the photocatalytic degradation of 1,2,4-triazole rings studied by Watanabe, the apparent nitrogen defect was related to the formation of organic nitrogen in the form of stable or low reactive compounds. By contrast, Guillard et al. reported for the photocatalytic degradation of other compounds with 1,2,4-triazole rings (4-hydroxy-1,2,4-triazolidine-3,5-dione, 4-phenyl-1,2,4-triazolidine-3,5-dione, 4-phenyl-1,2,4-triazole-2,5-dione) a conversion of about 17% of the organic nitrogen into N_2_ [50,52]. The most significant production under the photocatalytic condition of molecular nitrogen was observed by Waki and co-workers during the degradation of molecules with hydrazo groups where the photoconversion of organic nitrogen into N_2_ reached 70% [50,53].

The identification of the principal BTz intermediates was carried out analyzing the irradiated suspension by HPLC-ESI-HRMS/MS. The total ion current (TIC) chromatograms showed for each irradiation time only one peak, which corresponds to BTz (Figure S2). The extraction of the ESI-MS spectra of this peak showed a signal at *m/z* = 120.054 that corresponded to the exact mass of protonated BTz (C_7_N_3_H_8_, *m/z* = 120.056, Figure S3). The MS^2^ spectra of this compound (Figure S4) showed product ions derived from the sequential loss of N_2_ (*m/z* = 28.006) and HCN (*m/z* = 27.011) from the precursor molecule with the formation of the aziridinic cation and cyclopentadiene, respectively. Weiss and Reemtsma have previously proposed this fragmentation [3].

The identification of the intermediates required the extraction from the full mass spectrum of the accurate masses related to photo-formed compounds. Regarding the chromatograms, we obtained an XIC (extracted ion current) chromatogram where the single signal related to the parent compound *m/z* was present, and there were one or more XIC chromatogram(s) corresponding to the *m/z* signals of the accurate masses of the transformation products (see Supplementary Material). At retention times between 2 and 5.5 (degradation at pH 3), two peaks with *m/z* 136.048 and two peaks with *m/z* 152.043 appeared (Figure S2). These *m/z* ratios corresponded to two mono- and two bi-hydroxilated 1H-benzotriazole isomers. The preferential site of hydroxylation was the aromatic ring. It has been often reported that in the photocatalytic transformation of aromatic compounds, the primary intermediates are the hydroxylated products on the aromatic ring [54]. The more reactive positions of the benzotriazole system are the 5 and the 7, and this explains the formation of the two observed monohydroxylated isomers. The MS^2^ spectra of the monohydroxylated products showed two principal product ions at *m/z* equal to 108.042 and 80.047, corresponding to a first loss of N_2_ (*m/z* = 28.004) and a subsequent loss of CO (*m/z* = 27.995). The fragmentation was explained firstly with the formation of the aziridinic cation, its rearrangement to tropyl cation, and finally with the CO loss to form the stable pyridinic cation. In this case, we did not observe the loss of HCN, because the OH subsistent stabilized the six-membered ring [3]. At basic pH, no peaks in the XIC mode at *m/z* equal to 136 and 152 (mono and bihydroxylated BTz isomers) were detected. The first steps of degradation at pH 11 did not involve the benzenic ring, but rather the triazole ring. The degradation might start with a direct hole transfer with the formation of a radical on the nitrogen atom stabilized for resonance with the π system. No evidence of the presence of cyanuric acid among the principal BTz intermediates was obtained; the extraction of the related *m/z* ratio gave no signal also at high irradiation times. This confirms (i) the complete mineralization obtained during the photocatalytic process and (ii) the difference between the photocatalytic degradation of 1,2,3-triazole and 1,2,4-triazole derivates.

### 3.2. Tolyltriazole Photodegradation

**Figure 4:**
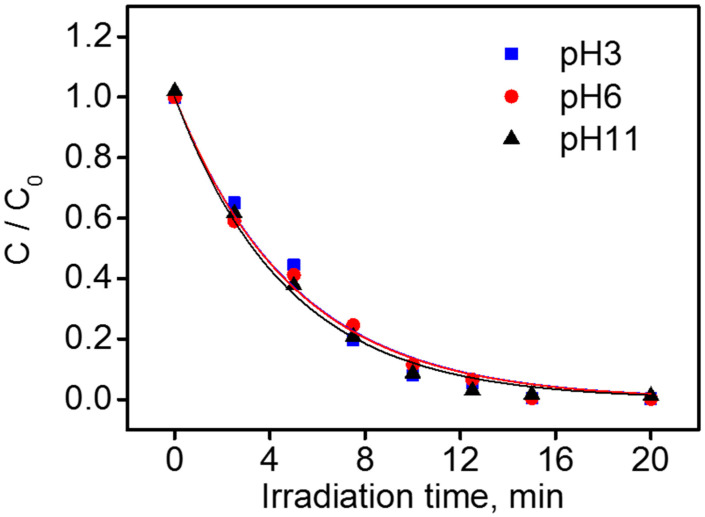
Tolyltriazole (TTz) photocatalytic degradation as a function of pH (conditions: TiO_2_ P25 0.5 g dm^−3^; TTz nominal initial concentration 1 × 10^−4^ M).

The trend of TTz disappearance under photocatalytic conditions is reported in Figure 4 at three different pHs. The initial degradation rates were 2.0 × 10^−2^, 2.00 × 10^−2^, and 2.11 × 10^−2^ at pH 3, 6, and 11, respectively.

The TTz degradation rate was almost unaffected by the pH. The tolyltriazole degradation rate was insensitive to the position of the valence band edge potential of TiO_2_, and this might be related with a scarce role of the direct hole transfer during the photocatalytic transformation of this substrate. Contrary to BTz, at basic pH, an increase of the TTz degradation rate was not observed. The pK_a_ of TTz is 8.8. At pH 11, the principal form of TTz is the deprotonated one. For TTz, the two species of the acid–base equilibrium showed a similar degradation rate. It was possible to infer that also at pH 11, the photocatalytic degradation might not involve the triazole ring at least for the first degradation steps (the portion involved in the acid–base equilibrium), but the aromatic ring (vide infra for the analysis of the principal intermediates).

**Figure 5:**
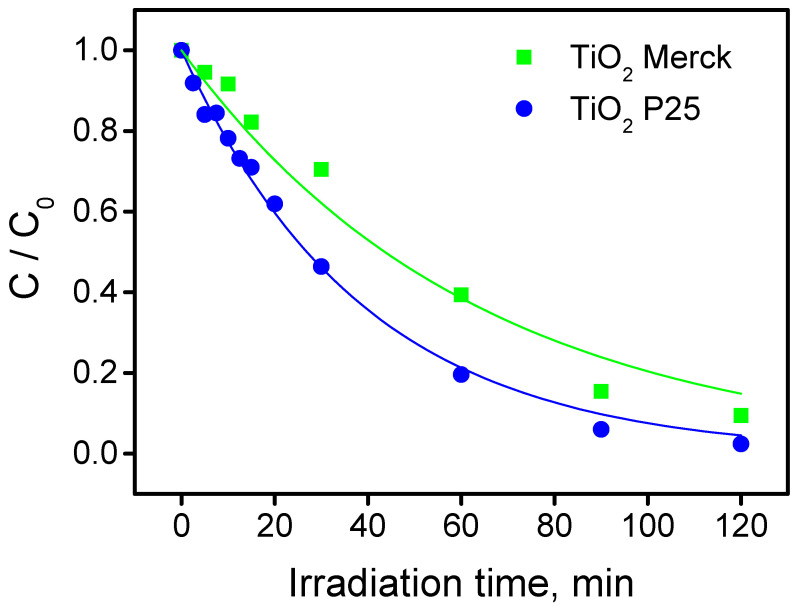
Tolyltriazole photodegradation profiles at pH 3 in the presence of TiO_2_ P25 and TiO_2_ Merck. (Conditions: TTz initial concentration 1 × 10^−3^ M, TiO_2_ concentration 0.5 g dm^−3^).

Figure 5 shows the time profiles of TTz disappearance over TiO_2_ P25 and Merck (initial TTz concentration 1 × 10^−3^ M, pH 3). The disappearance rates in the presence of P25 and Merck TiO_2_ were 2.58 × 10^−2^ and 1.6 × 10^−2^ mM min^−1^, respectively. The ratio rate_P25_/rate_Merck_ was 1.6. Values for this parameter near to 1 were observed with substrates (e.g., phenol) that are photocatalytically transformed mainly by HO^•^-like mediated oxidation. From the observed experimental evidences for the degradation of TTz, it was possible to infer that the contribution of this mediated oxidation was predominant compared to the direct hole transfer. Merck TiO_2_ has a specific surface area (SSA) equal to 10 m^2^ g^−1^ [35], which is one-fifth the P25 TiO_2_ SSA. The former showed an intrinsic activity (photocatalytic activity normalized per SSA) higher than that of P25 TiO_2_.

The identification of the first main tolyltriazole intermediates was carried out by HPLC-ESI-HRMS/MS (Figure S5). The TIC chromatograms showed for each irradiation time only one peak, which corresponds to TTz. The extraction of the ESI-MS spectra of this peak showed a signal at *m/z* = 134.078, which is the exact mass of protonated TTz (C_7_N_3_H_8_, *m/z* = 134.072). The XIC traces related to the *m/z* ratios of the hypothetical intermediates showed the photoformation of at least four monohydroxylated tolyltriazole isomers (recorded *m/z* 150.064, accurate *m/z* for C_7_N_3_OH_8_ 150.067) and three bihydroxylated isomers (recorded *m/z* 166.064, accurate *m/z* for C_7_N_3_O_2_H_8_ 166.062). The photoformation of the mono and bihydroxylated isomers was observed during all the tolyltriazole degradations. The change of the pH did not affect the nature of the transformation products. No change in the mechanism was proposed with the change of the pH. The formation of mono and bihydroxylated intermediates was explainable with the oxidation of the TTz aromatic ring (i.e., the substitution on a hydrogen atom with the OH group, whatever is the mechanism—mediated or direct—for the hole transfer).

The fragmentation of the TTz was monitored recording the MS^2^ spectra (precursor ion: 134 *m/z*, Figure S6). The protonated TTz lost subsequently a N_2_ molecule with the generation of a product ion with *m/z* 106.135 (134.138 − 106.135 = 28.003, the accurate mass of molecular nitrogen is 28.006) and a molecule of HCN with the formation of a product ion with *m/z* 79.103 (106.117 − 79.103 = 27.014, the exact mass of a HCN molecule is 27.011). The ESI–MS^2^ spectra of the monohydroxylated tolyltriazole isomers (precursor ion: 150 *m/z*, Figure S7) showed two different fragmentation pathways. The first started with a first loss of N_2_ with the formation of a product ion with *m/z* 122.132 (150.141 − 122.132 = 28.009, exact mass of N_2_ 28.006) and the subsequent loss of CO with the formation of the stable pyridinic cation with *m/z* equal to 94.122 (122.121 − 94.122 = 27.999, the accurate mass of CO is 27.995). The hydroxyl group on the aromatic ring stabilizing the six-membered ring avoided the loss of HCN and the consequence break of the six-membered ring. The second path started with the formation of a fragment with *m/z* 132.127 that corresponds to the loss of a molecule of water (150.141 − 132.127 = 18.014, the accurate mass of water is 18.020); subsequently, this fragment lost N_2_ and HCN, forming the cation with *m/z* equal to 77.147 (132.167 − 77.147 = 55.020, the sum of the accurate masses of N_2_ and HCN is 55.017). Differently from the first path, in this case, the loss of water removed the hydroxyl group from the aromatic ring, and so the fragmentation was equal to that observed for TTz.

Figure S1 shows the NPOC profile during the photocatalytic degradation of a 1 × 10^−3^ M TTz solution at pH 3. A complete mineralization was observed after 240 min. A monotonic straight profile was observed with a mineralization rate equal to 4.0 × 10^−3^ mM C min^−1^. The absence of intermediates stable under photocatalytic conditions makes the photocatalytic approach a useful tool for the complete abatement of TTz, avoiding its release in the environment.

**Figure 6:**
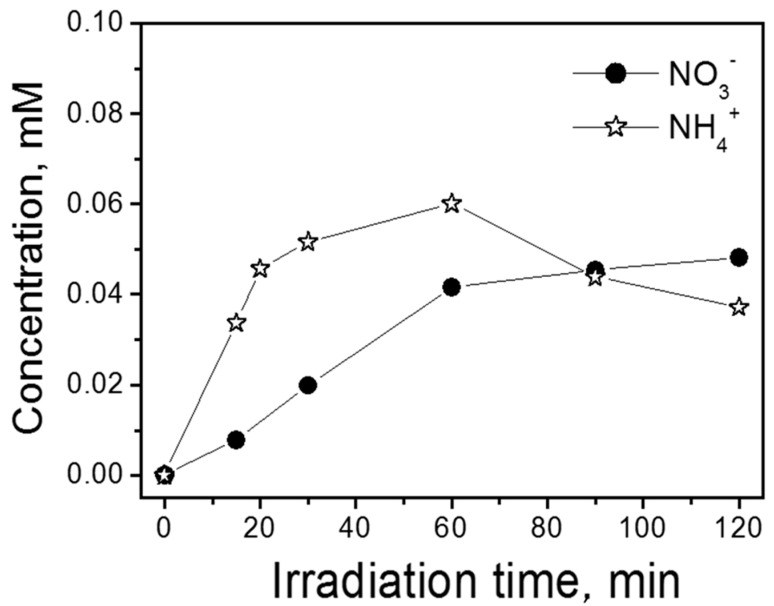
NO_3_^−^ and NH_4_^+^ concentration profile during the photocatalytic degradation of TTz at pH 6 (conditions: TTz initial concentration 1 × 10^−4^ M; TiO_2_ P25 0.5 g dm^−3^).

Figure 6 shows the time evolution of the ammonium and nitrate ion concentration during the photocatalytic degradation of TTz (1 × 10^−4^ M) at pH 6. The total mineralization of one TTz mole leads to 3 moles of inorganic nitrogen. The sum of NH_4_^+^ and NO_3_^−^ molar concentration released in solution after long irradiation reached a limiting value of one mole per mole of TTz converted. The conversion of the organic nitrogen to NH_4_^+^ and NO_3_^−^ was almost 33%. As previously outlined, the missing nitrogen might be assigned either to the formation of molecular nitrogen during the degradation of the 1,2,3-triazole ring, or to the formation of stable organic compounds containing nitrogen (e.g., cyanuric acid).

The mineralization of carbon was monitored measuring the evolution of Non Purgeable Organic Carbon (NPOC) concentration. As observed for BTz, a complete carbon mineralization of TTz was observed. This suggests that the missing nitrogen cannot be related to the release of stable organic nitrogen (i.e., cyanuric acid) during the TTz photocatalytic transformation. The NPOC went to zero, indicating that no organic nitrogen remained in solution. As a consequence, the balance of nitrogen can be closed only invoking the formation of N_2_. The structure of the 1,2,3-triazole ring with three nitrogen atoms directly bonded and the previously commented data about the loss of nitrogen during the BTz photocatalytic transformation were in agreement with the proposed pathways.

### 3.3. Tinuvin P Photodegradation

**Figure 7:**
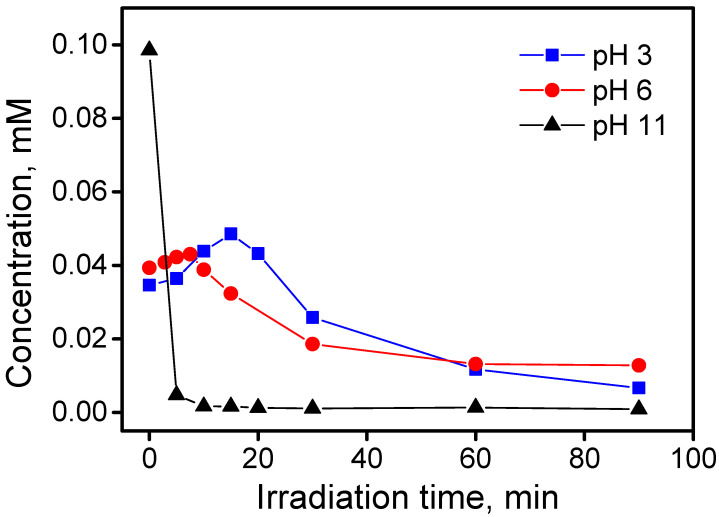
Tinuvin P photocatalytic degradation as a function of pH (conditions: Tinuvin P initial concentration 1 × 10^−4^ M; TiO_2_ P25 0.5 g dm^−3^; solvent CH_3_CN/H_2_O 80/20).

The last substrate subjected to photocatalytic degradation was 2-(2H-benzotriazol-2-yl)-p-cresol, which was commercialized with the name Tinuvin P (TP). TP shows low water solubility (0.173 mg dm^−3^), and so the photocatalytic tests were carried out in a low-polarity medium (acetonitrile/water mixture). Figure 7 shows the photocatalytic degradation profiles for the disappearance of Tinuvin P (1 × 10^−4^ M) in CH_3_CN/Water 80:20 at different pH values.

Before irradiation, the nominal concentration was observed in solution only at pH 11. At pH 3 and 6, despite the different medium adopted (CH_3_CN/Water 80:20), the solutions were presumably saturated, and part of TP was precipitated on the TiO_2_ surface. At pH 11, the partial deprotonation of the phenolic group negatively charged the molecules and increased the TP solubility in polar solvents. The initial degradation rate at pH 11 in this condition was 6.0 × 10^−2^ mM min^−1^. The change of the used medium hindered quantitative comparison of the Tinuvin P degradation rate with the BTz and TTz transformation rates reported above. At pH 3 and 6, the initial concentration in solution was 0.04 mM. In the first 10 min of irradiation, we did not observe a significant decrease of the TP concentration; on the contrary, a slightly increase was observed. It was possible to suppose that (i) the decrease of the TP concentration in solution, (ii) a slight increase of the temperature under irradiation and (iii) the photocatalytic degradation of the first layer of the precipitated TP at the surface promoted its dissolution. Furthermore, the irradiation might activate photodesorption phenomena that allowed the partial solubilization of the TP adsorbed at the surface. As observed above for the photocatalytic degradation of BTz, the maximum in the TP disappearance rate was at pH 11: the photoproduced oxidant species oxidized the phenate anion easier than the phenol moiety. This experimental evidence suggested that the first steps of the Tinuvin P degradation could involve not the benzotriazole unit, but the phenol.

From the HPLC-ESI-HRMS/MS analysis, it was possible to identify the nature of the most important intermediates (Figures S8–S10). A relevant accumulation of 1H-benzotriazole (*m/z* = 120.055) during the analysis was observed. Five hydroxylated derivates of Tinuvin P were detected; among these, three mono isomers (*m/z* = 242.094) and two bihydroxylated isomers (measured *m/z* = 258.087) were identified. From the analysis of the MS^2^ spectra of the hydroxylated derivates, it was possible to identify the preferential site of hydroxylation. The MS^2^ spectra of all these intermediates generated a product ion with an *m/z* equal to 120.055, which is the *m/z* of the protonated BTz. This confirmed that the first preferential site of hydroxylation was not the benzotriazole system, but rather the phenolic ring. In the MS^2^ spectra, we did not observed any fragments with *m/z* equal to that of the mono or bihydroxilated BTz. This was in agreement with the higher oxidability of the phenolic ring with respect to 1H-benzotriazole with a deactivated aromatic ring.

A relevant accumulation of 1H-benzotriazole during the analysis was observed. Figure S11 shows the disappearance profile of TP and the concomitant accumulation of BTz during the photocatalytic degradation of Tinuvin P (1 × 10^−4^ M) in CH_3_CN/Water 90:10 at pH 3. The main product of the photocatalytic degradation of Tinuvin P is 1H-benzotriazole; presumably, this molecule would follow the degradation pathways outlined above until achieving a complete mineralization.

## 4. Conclusions

The photogenerated species at the semiconductor/electrolyte interface lead to a rapid conversion of the investigated benzotriazoles substrates. Maximum disappearance rates equal to 3.9 × 10^−2^, 2.1 × 10^−2^, and 6.0 × 10^−2^ mM min^−1^ were observed for 1H-benzotriazole, tolyltriazole, and Tinuvin P, respectively. The photocatalytic degradation gave a complete mineralization of the substrates (BTz and TTz), while no accumulation of persistent photostable intermediates was observed.

The nitrogen mass balance for the photodegradation of BTz and TTz showed a loss of nitrogen presumably related to the formation of molecular nitrogen. This can be related to the presence of three contiguous nitrogen atoms in the benzotriazoles triazole ring. The formation of N_2_ was also proposed to explain the scarce recovery observed in the total nitrogen (TN) analysis of solutions of the same substrates through high-temperature catalytic oxidation (HTCO). In this case, the conversion of the organic nitrogen to the detectable NO was partially limited by the formation of molecular nitrogen [55].

The ubiquitous presence of BTz and TTz in aquatic environmental systems has been reported and related to the inability of the common WWTPs (Wastewater Treatment Plant) to degrade these compounds. The photocatalytic process under irradiated TiO_2_ and other Advanced Oxidation Processes (AOPs) that use HO^•^ as the main oxidant might be useful tools for the abatement of these CECs from both the effluents of WWTPs and the raw waters used for drinking water production. Further investigation is needed to highlight strengths and weaknesses and to demonstrate the applicability of the photocatalytic technology for the abatement of benzotriazoles in real-world contexts.

## Supplementary Materials

The following are available online at https://www.mdpi.com/2079-4991/10/9/1835/s1. Table S1: Main chemical characteristics of the compounds. Table S2: Initial degradation rates for the photocatalytic degradation of 1H-benzotriazole and tolyltriazole 1 × 10^−4^ M at different pHs. Figure S1: Non Purgeable Organic Carbon (NPOC) evolution during the photocatalytic degradation of 1H-benzotriazole and Tolyltriazole at pH 3. Figure S2: ESI–HR chromatograms of a 1H-benzotriazole suspension irradiated under photocatalytic conditions. Figure S3: ESI–HRMS spectra of 1H-benzotriazole. Figure S4: ESI–MS^2^ spectra of 1H-benzotriazole (precursor ion: 120 *m/z*). Figure S5: ESI–HRMS chromatograms of a tolyltriazole suspension irradiated under photocatalytic conditions. Figure S6: (a) ESI–HRMS spectra of the tolyltriazole peak, (b) ESI–MS^2^ spectra of tolyltriazole (precursor ion: 134 *m/z*). Figure S7: (a) ESI–HRMS spectra of the peak at retention time = 13.49 min. (b) ESI–MS^2^ spectra of the same monohydroxylated tolyltriazole isomer (precursor ion: 150 *m/z*). Figure S8: ESI–HRMS chromatograms of a Tinuvin P suspension irradiated under photocatalytic conditions. Figure S9: ESI–HRMS spectra of the peak at retention time (a) 9.51 min and (b) 10.45 min. Figure S10: ESI–MS^2^ spectra of (a) a monohydroxylated Tinuvin P isomer (precursor ion: 242 *m/z*), (b) a bihydroxylated Tinuvin P isomer (precursor ion: 258 *m/z*). Figure S11: Tinuvin P and 1H-benzotriazole concentration time profile during the Tinuvin P photodegradation at pH 3.
