# Supplementary material for: Photocatalytic Transformations of 1H-Benzotriazole and Benzotriazole Derivates
Source: Nanomaterials (Basel). 2020 Sep 14;10(9):1835. doi: 10.3390/nano10091835 (PMC7560172; doi:10.3390/nano10091835)
Supplement: Supplementary file 1 [file nanomaterials-10-01835-s001.pdf]

Photocatalytic Transformations of 1H-Benzotriazole and Benzotriazole Derivates

Marco Minella [1], Elisa De Laurentiis [1], Francesco Pellegrino [1,2], Marco Prozzi [1], Federica Dal Bello [3], Valter Maurino [1,2,*] and Claudio Minero [1]

[1] Chemistry Department and NIS Interdepartmental Centre, University of Torino, Via P. Giuria 5, Turin 10125, Italy; marco.minella@unito.it (M.M.); elisa.delaurentiis@tiscali.it (E.D.L.); francesco.pellegrino@unito.it (F.P.); marco.prozzi@unito.it (M.P.); claudio.minero@unito.it (C.M.)
[2] JointLAB UniTo-ITT Automotive, Via Quarello 15/A, 10135 Torino, Italy
[3] Department of Molecular Biotechnology and Health Sciences, University of Torino, Via P. Giuria 5, Turin 10125, Italy; federica.dalbello@unito.it
* Correspondence: valter.maurino@unito.it; Tel.: +39-011-6705218.

Supplementary Materials

Table 1. Main chemical and physical characteristics of the three compounds considered.

Name	1H-benzotriazole	Tolyltriazole	Tinuvin P
Chemical Formula	$C_6H_5N_3$	$C_7H_7N_3$	$C_{13}H_{11}N_3O$
Molecular Mass, u	119.05	133.06	225.09
Solubility in water (25°C) (around neutrality)	28 g dm^{-3}	7 g dm^{-3}	< 0.01 g dm^{-3} (soluble in basic media)
pKa	8.6	8.8	/
LogK_{ow} (octanol-water)	1.23	1.89	4.31

Table S2. Initial degradation rates for the photocatalytic degradation of 1H-benzotriazole and tolyltriazole 1×10^{-4} M at different pHs (TiO_2 P25 0.5 g dm^{-3}). The error bars are computed as the fitting error of the experimental profiles obtained from the replicates.

	Degradation rate, mM min^{-1}	
	1H-benzotriazole	Tolyltriazole
pH 3	(2.27±0.09)×10^{-2}	(2.0±0.1)×10^{-2}
pH 6	(1.2±0.1)×10^{-2}	(2.00±0.08)×10^{-2}
pH 11	(3.88±0.05)×10^{-2}	(2.11±0.09)×10^{-2}

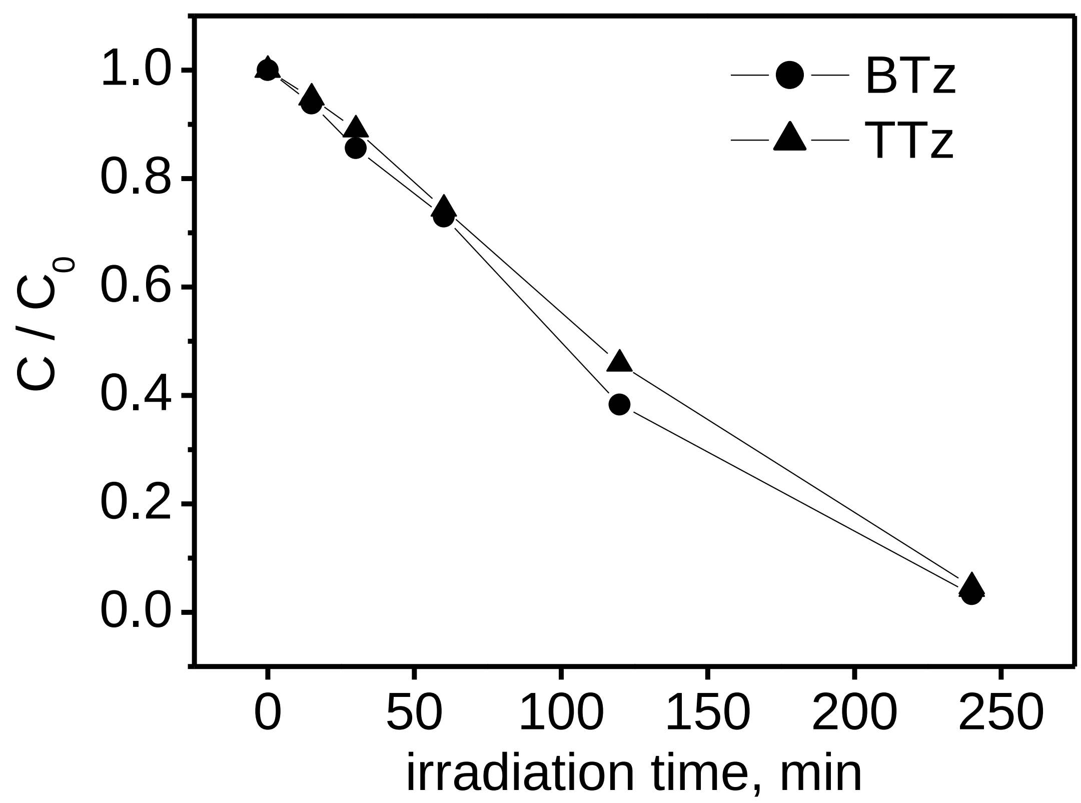

Figure S1. Non Purgeable Organic Carbon (NPOC) evolution during the photocatalytic degradation of 1H-benzotriazole and tolyltriazole at pH 3 (Conditions: BTz and TTz nominal initial concentration 1×10^{-3} M; TiO_2 P25 0.5 g dm^{-3}).

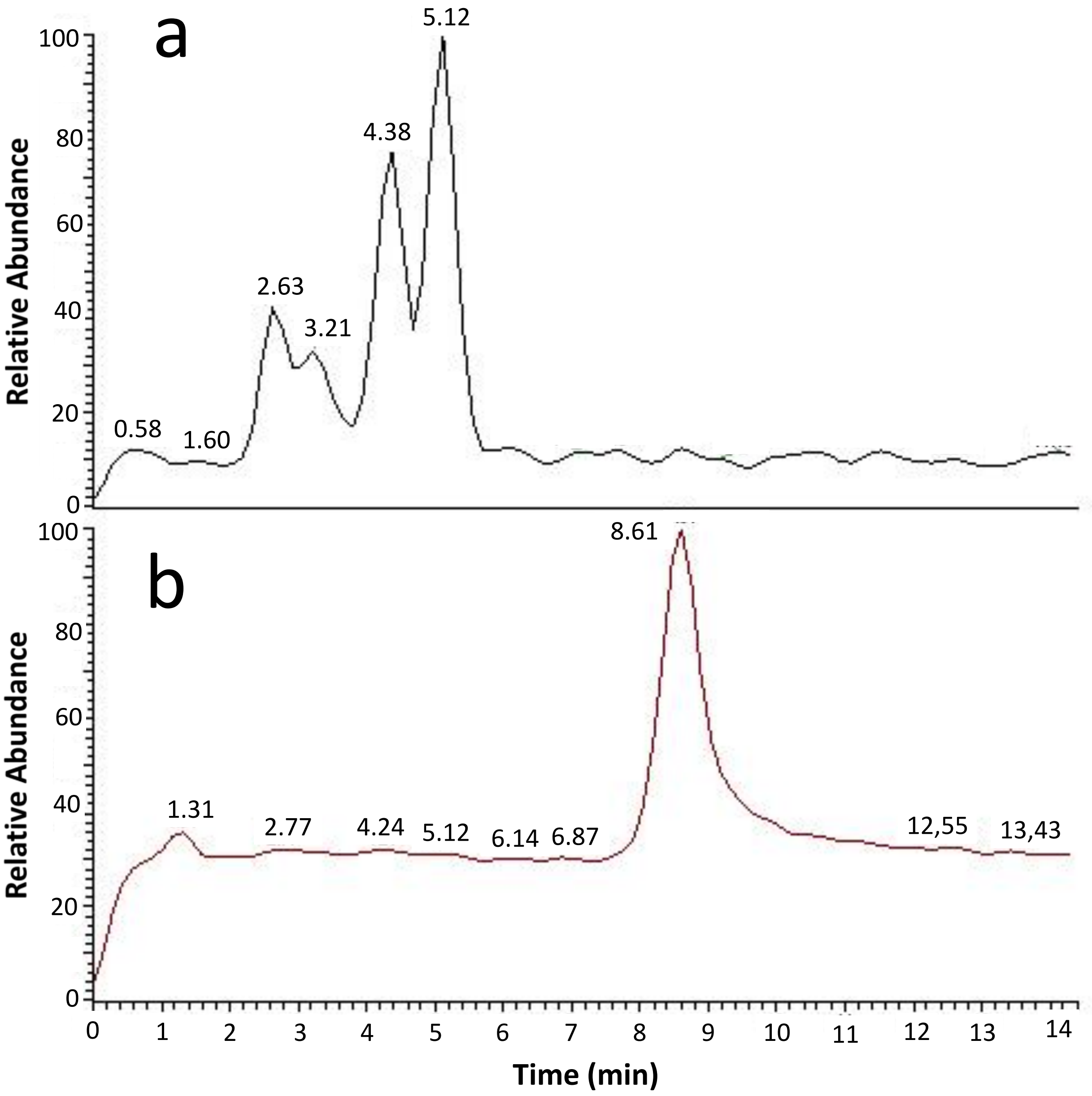
a
5.12
4.38
2.63
3.21
0.58
1.60
Relative Abundance
100
80
60
40
20
0
b
8.61
1.31
2.77
4.24
5.12
6.14
6.87
12,55
13,43
Relative Abundance
0 1 2 3 4 5 6 7 8 9 10 11 12 13 14
Time (min)

Figure S2. ESI–HRMS chromatograms of a 1H–benzotriazole suspension irradiated under photocatalytic conditions (conditions: initial nominal concentration 5.0×10^{-4} M, irradiation time 15 minutes, pH 3). Lower trace: total ion current (TIC) chromatogram, note the only presence of the peak related to BTz (t_r = 8.6 minutes); upper trace: extracted chromatogram (XIC) related to the *m*/*z* equal to 136 and 152 (mono and bihydroxylated BTz intermediates).

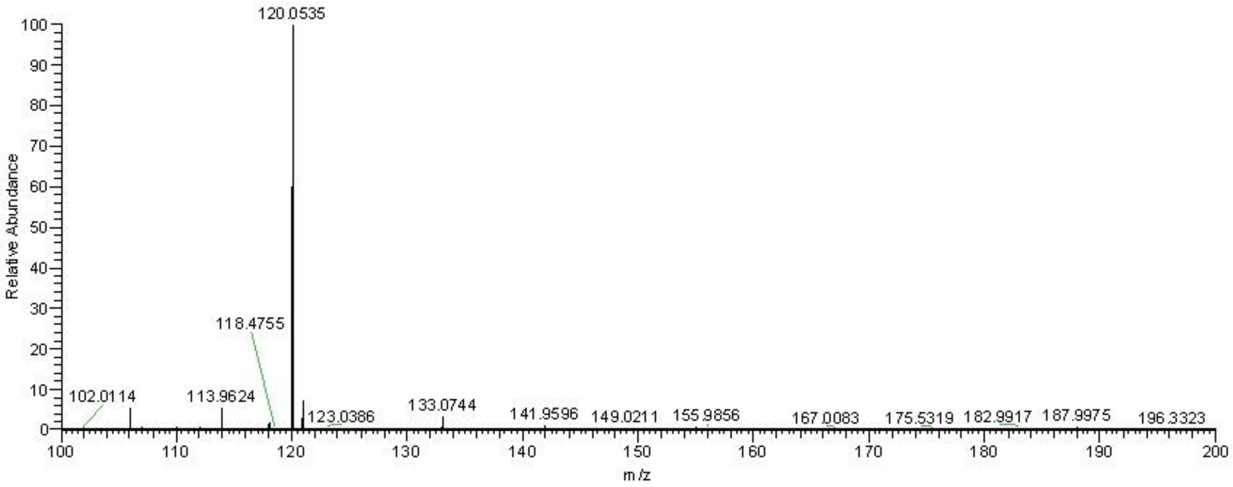

Figure S3. ESI–HRMS spectra of 1H-benzotriazole. Note the signal at *m*/*z*= 120.0535 corresponds to the accurate mass, within the experimental error, of protonated BTz ($C_6N_3H_5$, *m*/*z*= 120.056).

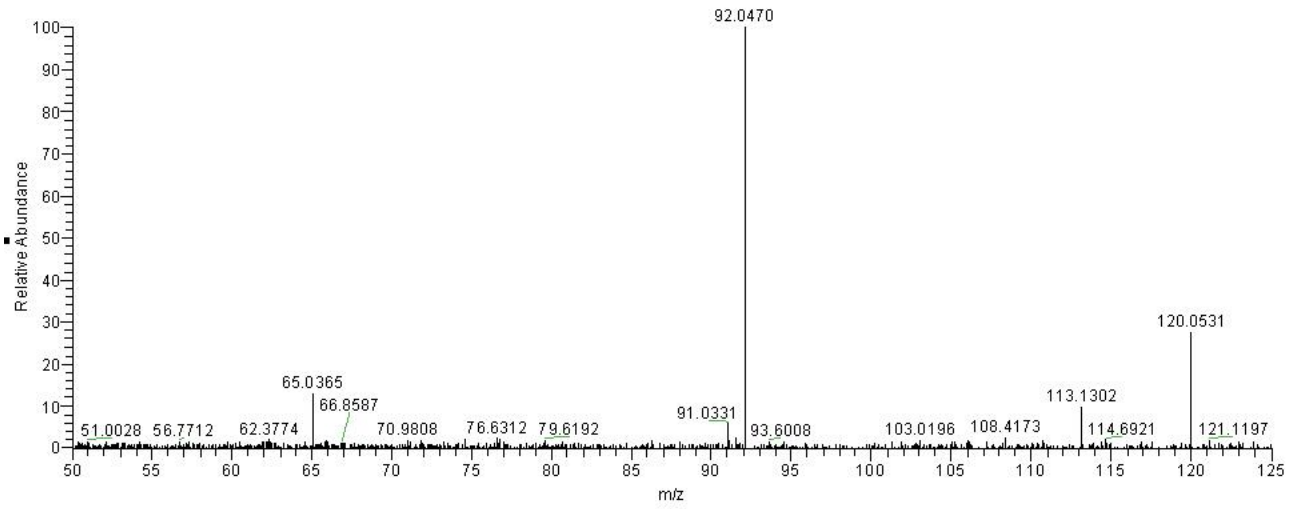

Figure S4. ESI–MS² spectra of 1H–benzotriazole (precursor ion: 120 *m*/*z*). Note the formation of a product ion with *m*/*z* 92.047 that corresponds to the loss of a N_2 molecule (120.053–92.047= 28.006, accurate mass of molecular nitrogen). The loss of a HCN molecule from the product ion with *m*/*z* 92.047 generates the fragment with *m*/*z* 65.037 (92.047 – 65.037 = 27.010, the accurate mass of a HCN molecule is 27.0110).

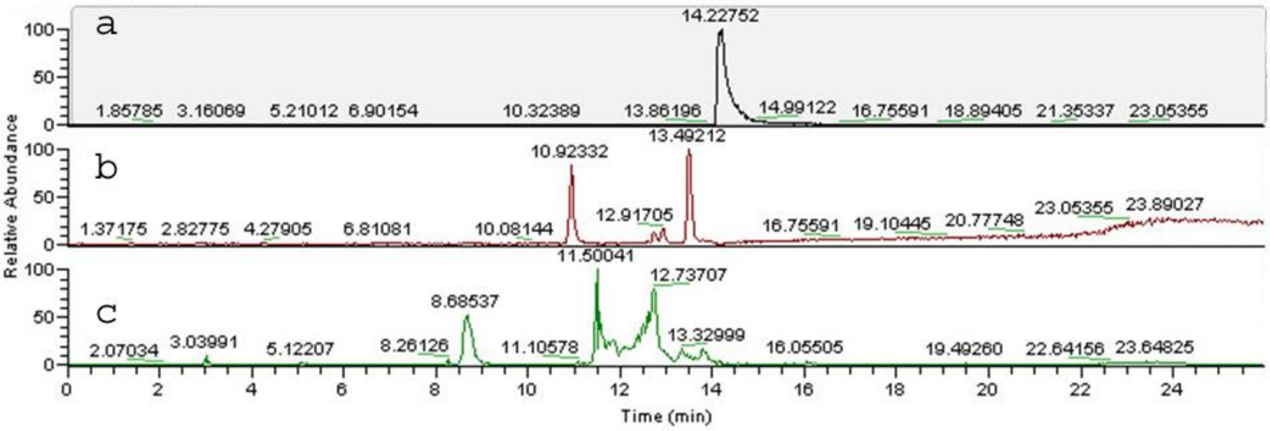

Figure S5. ESI–HRMS chromatograms of a tolyltriazole suspension irradiated under photocatalytic conditions (conditions: initial nominal concentration 1.0×10^{-4} M, irradiation time 30 minutes, pH 6). a) XIC for *m*/*z* 134.072, note the only presence of the peak related to TTz (t_r = 14.22 minutes); b) XIC for *m*/*z* 150.067 (hydroxylated TTz isomers mass, note the presence of at least four isomers; c) XIC for *m*/*z* 166.062 (bihydroxylated TTz isomers mass), note the presence of at least three different isomers.

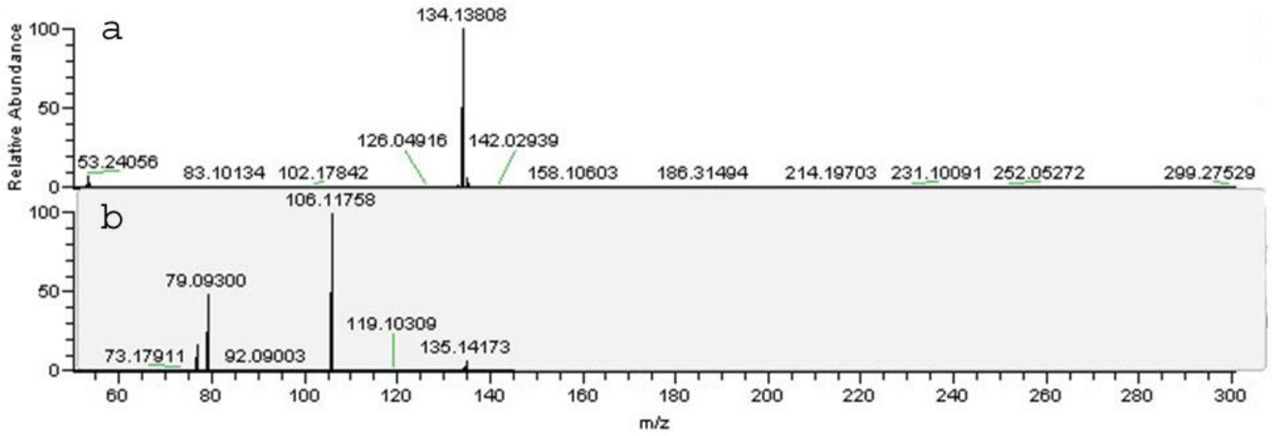

Figure S6. a) ESI–HRMS spectra of the tolyltriazole peak. Note the signal at *m*/*z*= 134.138 that corresponds to the accurate mass of protonated TTz ($C_7N_3H_8$, *m*/*z*= 134.072). b) ESI–MS^2 spectra of tolyltriazole (precursor ion: 134 *m*/*z*). Note the formation of a product ion with *m*/*z* 106.117 that corresponds to the loss of a N_2 molecule (134.1381–106.1176 = 28.0205, the accurate mass of molecular nitrogen is 28.006). The loss of a HCN molecule from the product ion with *m*/*z* 106.117 generates another product ion with *m*/*z* 79.093 (106.118 – 79.093 = 27.025, the exact mass of a HCN molecule is 27.011).

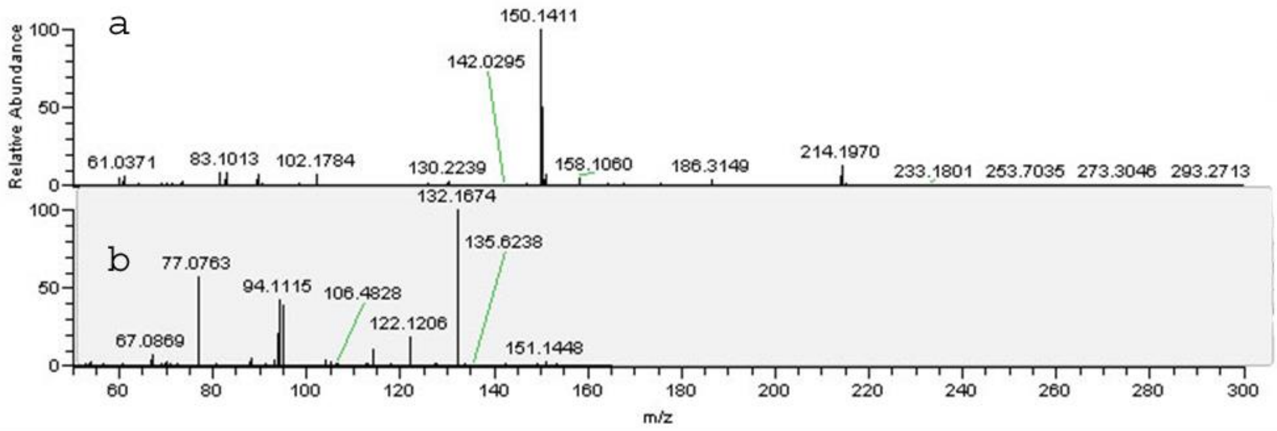

Figure S7. a) ESI–HRMS spectra of the peak at retention time = 13.49 min. Note the signal at *m/z*= 150.1411 that corresponds to the accurate mass of a monohydroxylated TTz isomer ($C_7N_3OH_8$, *m/z*= 150.067). b) ESI–MS^2 spectra of the same monohydroxylated tolyltriazole isomer (precursor ion: 150 *m/z*). Note the formation of a product ion with *m/z* 132.167 that corresponds to the loss of a H_2O molecule (150.141–132.167 = 17.974, the accurate mass of water is 18.020). The fragment with *m/z* 122.121 was formed after the loss of a N_2 molecule from the precursor ion (150.141-122.121=28.030, exact mass of N_2 28.006). The loss of CO from the 122.121 ion generates the product ion with *m/z* equal to 94.112 (122.121 – 94.112 = 28.009, the accurate mass of CO is 27.995). The product ion with *m/z* 77.076 is formed from the successive loss of N_2 and HCN from the ion with *m/z* equal to 132.167 (132.167–77.076=55.091, the sum of the accurate masses of N_2 and HCN is 55.017).

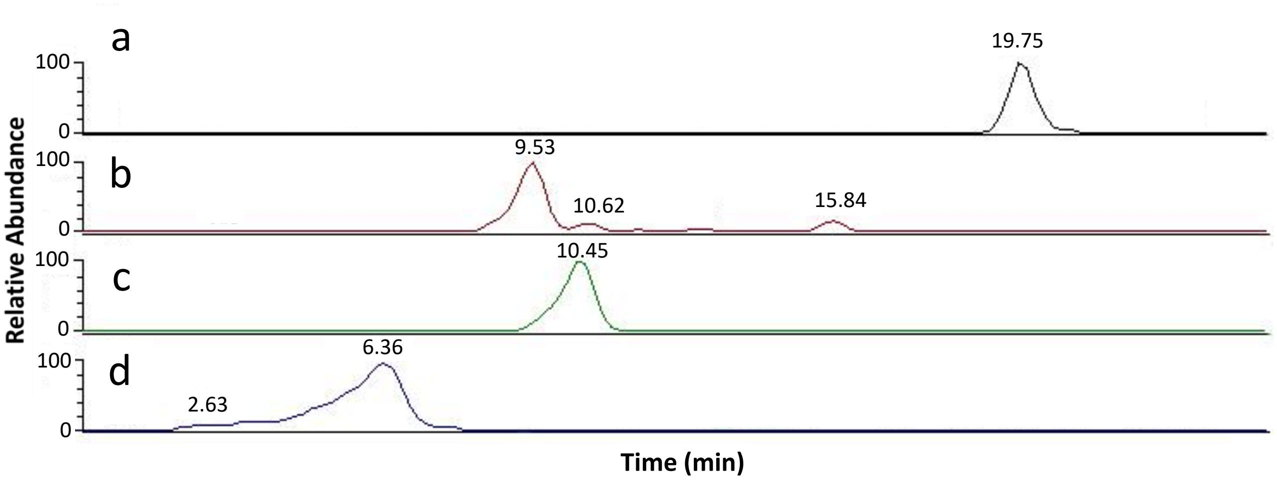

Figure S8. ESI–HRMS chromatograms of a Tinuvin P suspension irradiated under photocatalytic conditions (conditions: initial nominal concentration 1.0×10^{-4} M, irradiation time 60 minutes, pH 3, solvent acetonitrile:water 90:10). a) XIC for *m*/*z* 226.098 (m/z protonated Tinuvin P, retention time 19.75 minutes); b) XIC for *m*/*z* 242.093 (hydroxylated TP isomers mass); c) XIC for *m*/*z* 258.088 (bihydroxylated TP isomers mass); d) XIC for *m*/*z* 120 (1H-benzotriazole).

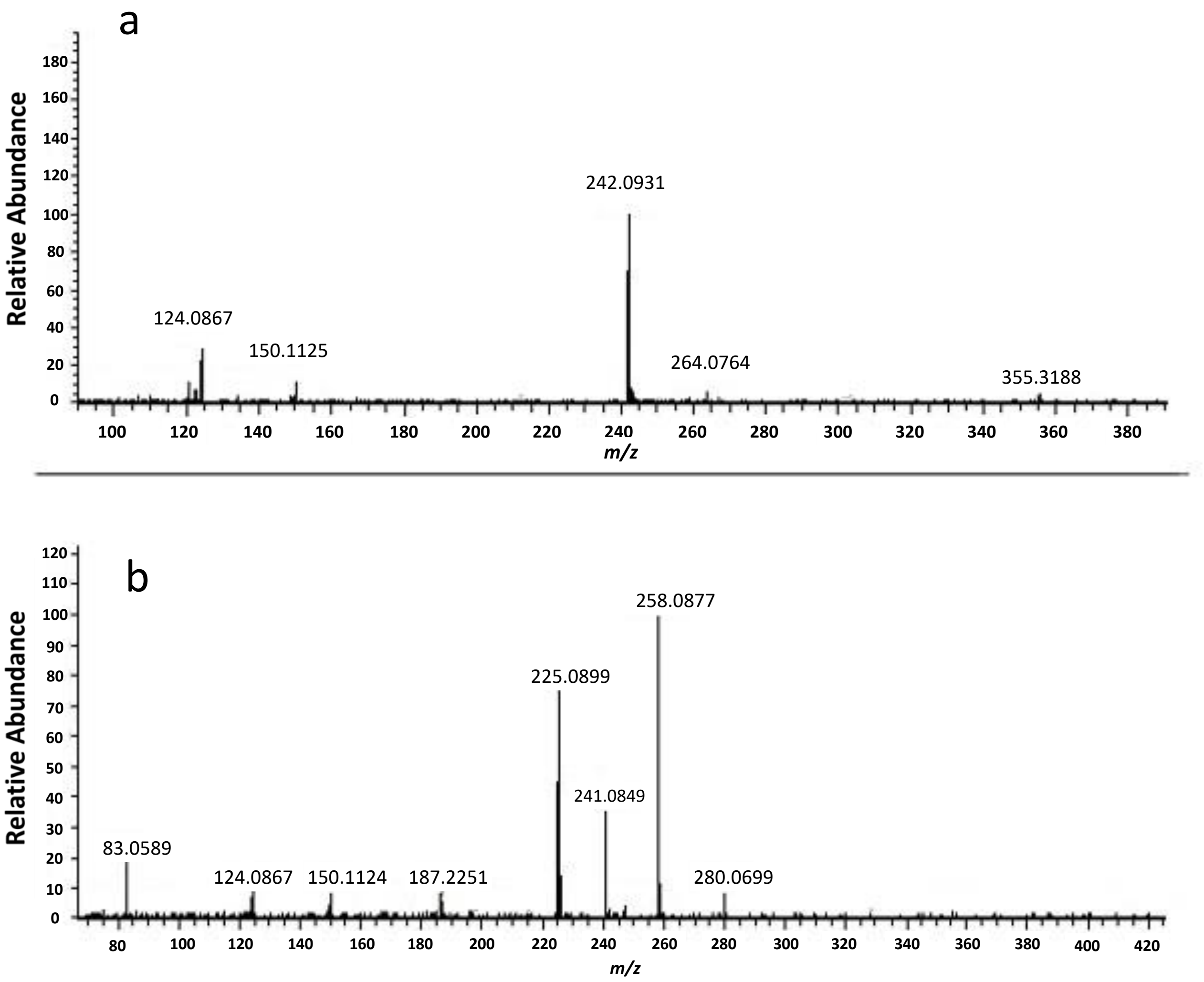

Figure S9. ESI–HRMS spectra of the peak at retention time a) 9.51 minutes and b) 10.45 minutes. Note the signals at *m/z*= 242.093 and *m/z*= 258.088 that correspond to the accurate mass of a monohydroxylated TP isomer ($C_{13}H_{12}N_3O_2$, *m/z*= 242.093) and of a bihydroxylated TP isomer ($C_{13}H_{12}N_3O_3$, *m/z*= 258.088) respectively.

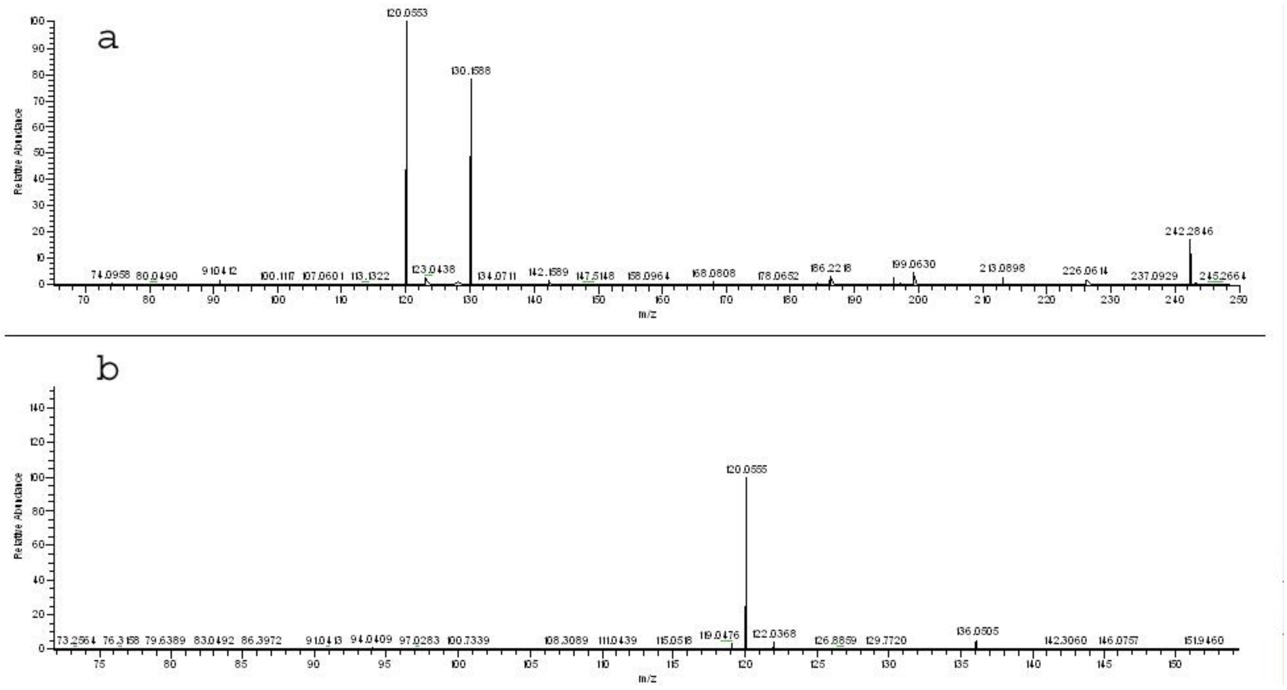

Figure S10. ESI–MS^2 spectra of a) a monohydroxylated Tinuvin P isomer (precursor ion: 242 *m/z*); b) a bihydroxylated Tinuvin P isomer (precursor ion: 258 *m/z*). Note in each case the presence of a product ion with *m/z* 120.056 that corresponds to the mass of 1H-benzotriazole ($C_6N_3H_5$, m/z = 120.056).

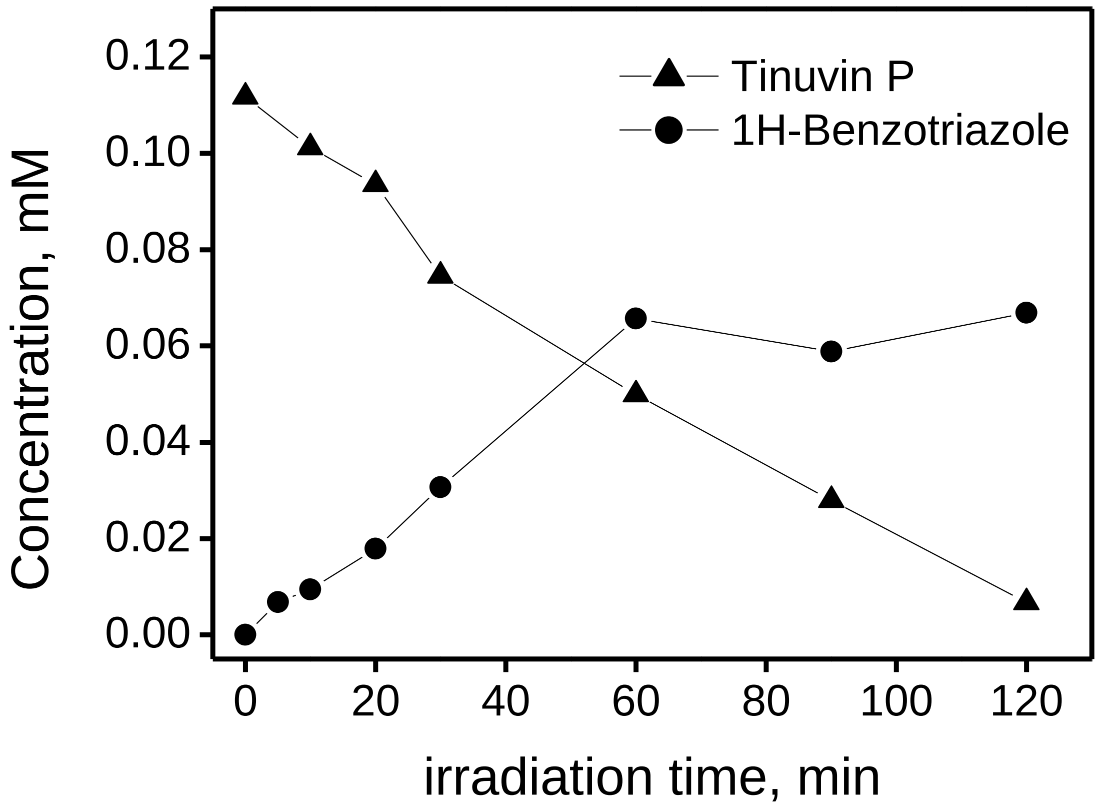

Figure S11. Tinuvin P and 1H-benzotriazole concentration time profile during the Tinuvin P photodegradation at pH 3 (Conditions: Tinuvin P initial concentration 1×10^{-4} M, Solvent CH_3CN/H_2O 90/10, TiO_2 Degussa P25 0.5 g dm^{-3})
